# Sulfate-bridged dimeric trinuclear copper(II)–pyrazolate complex with three different terminal ligands

**DOI:** 10.1107/S2056989016010719

**Published:** 2016-07-08

**Authors:** Gellert Mezei

**Affiliations:** a Department of Chemistry, Western Michigan University, Kalamazoo, Michigan, USA

**Keywords:** crystal structure, copper, pyrazolate, trinuclear complex, terminal sulfate ligand

## Abstract

The reaction of CuSO_4_·5H_2_O, 4-chloro­pyrazole (4-Cl-pzH) and tri­ethyl­amine (Et_3_N) in di­methyl­formamide (DMF) produced crystals of di­aqua­hexa­kis­(μ-4-chloro­pyrazolato-κ^2^ *N*:*N*′)bis­(*N*,*N*-di­methyl­formamide)di-μ_3_-hydroxido-bis­(μ_4_-sulfato-κ^4^ *O*:*O*′:*O*′′:*O*′′)hexa­copper(II) *N*,*N*-di­methyl­formamide tetra­solvate dihydrate, [Cu_3_(OH)(SO_4_)(C_3_H_2_ClN_2_)_3_(C_3_H_7_NO)(H_2_O)]_2_·4C_3_H_7_NO·2H_2_O. The centrosymmetric dimeric molecule consists of two trinuclear copper–pyrazolate units bridged by two sulfate ions. The title compound provides the first example of a trinuclear copper–pyrazolate complex with three different terminal ligands on the Cu atoms, and also the first example of such complex with a strongly binding basal sulfate ion. Within each trinuclear unit, the Cu^II^ atoms are bridged by μ-pyrazolate groups and a central μ_3_-OH group, and are coordinated by terminal sulfate, H_2_O and DMF ligands, respectively. Moreover, the sulfate O atoms coordinate at the apical position to the Cu atoms of the symmetry-related unit, providing square–pyramidal coordination geometry around each copper cation. The metal complex and solvent mol­ecules are involved in O—H⋯O hydrogen bonds, leading to a two-dimensional network parallel to (10-1).

## Abstract 2

The crystal structure of the first trinuclear copper–pyrazolate (pz) complex with three different terminal ligands, including a strongly binding sulfate, is reported. The sulfate ion also acts as bridging ligand, leading to a dimeric structure, [Cu_3_(μ_3_-OH)(μ-4-Cl-pz)_3_(μ_4_-SO_4_)(DMF)(H_2_O)]_2_·4DMF·2H_2_O.

## Chemical context   

Trinuclear copper(II) complexes are primarily studied for their relevance to multicopper enzymes, such as oxidases (*e.g.*, laccase, ascorbate oxidase, ceruloplasmin), oxygenases (*e.g.*, tyrosinase, particulate methane monooxygenase, ammonia monooxygenase) and reductases (*e.g.*, nitrite reductase, nitrous oxide reductase) (Solomon *et al.*, 1996 ▸, 2014 ▸). Thus, such complexes are important targets from synthesis, redox chemistry and catalysis viewpoints (Di Nicola *et al.*, 2009 ▸; Mimmi *et al.*, 2004 ▸; Tsui *et al.*, 2011 ▸; Lionetti *et al.*, 2013 ▸; Grundner *et al.*, 2015 ▸). Trinuclear copper(II) complexes also display inter­esting spectroscopic and magnetic properties (Boča *et al.*, 2003 ▸; Rivera-Carrillo *et al.*, 2008 ▸; Spielberg *et al.*, 2015 ▸), and have been crucial in studying concepts such as spin frustration (Fu *et al.*, 2015 ▸). The pyrazolate anion is an excellent ligand for the construction of cyclic trinuclear and higher nuclearity metal complexes, leading to a variety of mol­ecular architectures based on copper or other metals (Halcrow, 2009 ▸; Viciano-Chumillas *et al.*, 2010 ▸).

A unique class of copper–pyrazolate complexes is defined by nanojars, based on a series of cyclic polymerization isomers, [*cis*-Cu^II^(μ-OH)(μ-pz)]_*n*_ (pz = pyrazolate anion, *n* = 6–14, except 11), which incarcerate anions with large hydration energies (*e.g.*, sulfate, phosphate, carbonate) with unprecedented strength (Fernando *et al.*, 2012 ▸; Mezei, 2015 ▸; Ahmed, Szymczyna *et al.*, 2016 ▸) and permits the extraction of such anions from water into aliphatic solvents (Ahmed, Calco *et al.*, 2016 ▸). Nanojars are obtained by self-assembly from a copper salt, pyrazole and a base (needed both for deprotonating pyrazole and as a hydroxide ion source) in the presence of an anion with large hydration energy, *via* a trinuclear inter­mediate, which is isolable and can be converted into nanojars by adding a base (Ahmed & Mezei, 2016 ▸). Use of a strong base, such as sodium or tetra­butyl­ammonium hydroxide, allows the preparation of nanojar solutions in different organic solvents. In contrast, a weak base, such as tri­ethyl­amine, can only be employed as hydroxide source (Et_3_N + H_2_O ↔ Et_3_NH^+^ + HO^−^) if the nanojar product is precipitated out of the solution by dilution with excess water, in which the nanojar is not soluble (Fernando *et al.*, 2012 ▸). Isolation of the title compound provides further evidence that in a neat organic solvent, such as *N*,*N*-di­methyl­formamide, the self-assembly process using tri­ethyl­amine halts at the trinuclear stage, due to the acidity of the conjugate acid (tri­ethyl­ammonium cation, p*K*_a_ = 10.75 in H_2_O).
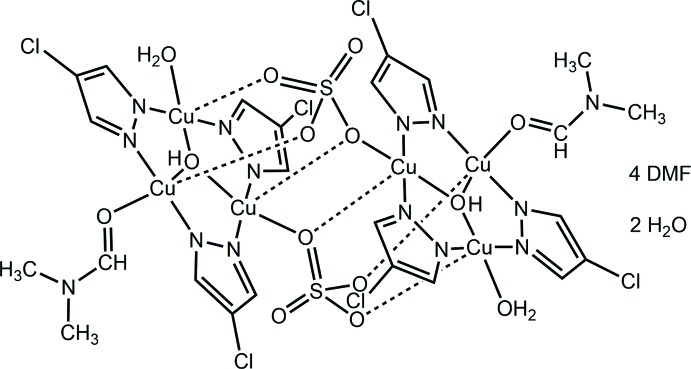


## Structural commentary   

**Figure 1:**
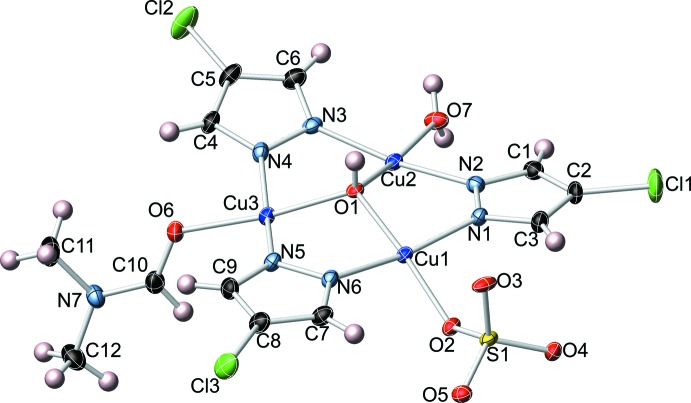
Displacement ellipsoid plot (50% probability level) of the asymmetric unit of the title complex, showing the atom-labeling scheme (DMF and H_2_O solvent mol­ecules omitted).

**Figure 2:**
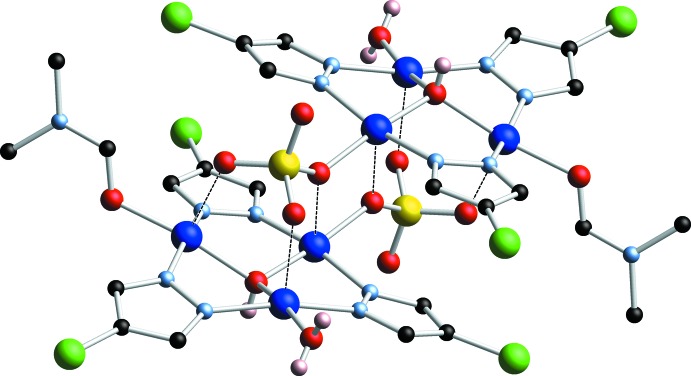
Dimeric structure formed by mutual apical coordination of three sulfate O atoms to the Cu atoms of the symmetry-related trinuclear copper(II)–pyrazolate complex.

The title metal complex mol­ecule, located around an inversion center, consists of two symmetry-related trinuclear copper pyrazolate units (Fig. 1 ▸) connected together by sulfate ions (Fig. 2 ▸). One O atom of the sulfate moiety coordinates to one of the three independent Cu^II^ atoms as basal donor [Cu1—O2: 1.976 (2) Å], and to the corresponding symmetry-related Cu^II^ atom as apical donor [Cu1′—O2: 2.277 (2) Å]. The other two O atoms of the sulfate moiety coordinate apically to the other two Cu atoms of the symmetry-related trinuclear unit, whereas the fourth O atom accepts a hydrogen bond from the solvent water mol­ecule (Table 1 ▸). A square–pyramidal coordination geometry around each of the Cu^II^ atoms is completed by the bridging μ-pyrazolate and μ_3_-OH moieties, and terminal water or di­methyl­formamide mol­ecules in basal positions. The Cu_3_(μ-4-Cl-pz)_3_ core is relatively flat, with dihedral angles between the 4-chloro­pyrazolate mean planes and the Cu_3_ mean plane of 1.74 (6), 7.20 (6) and 14.10 (4)°. The μ_3_-OH group is located 0.5615 (15) Å above the Cu_3_ mean plane. Bond lengths and angles within the Cu_3_(μ-4-Cl-pz)_3_ framework are similar to the ones found in related complexes (Mezei *et al.*, 2007 ▸; Rivera-Carrillo *et al.*, 2008 ▸). The sulfate-bridged dimeric structure presented here is reminiscent of dimeric trinuclear copper–pyrazolate complexes with bridging carboxyl­ates (Mezei *et al.*, 2004 ▸; Casarin *et al.*, 2005 ▸).

## Supra­molecular features   

The dimeric metal complex participates in an intricate hydrogen-bond network with the solvent DMF and H_2_O mol­ecules. Numerical details of the hydrogen bonding are given in Table 1 ▸. The μ_3_-OH group donates a hydrogen bond to a solvent DMF mol­ecule [O1⋯O9: 2.711 (3) Å], whereas the coordinating water mol­ecule donates two hydrogen bonds, one to the solvent water mol­ecule [O7⋯O10: 2.625 (3) Å] and one to the other independent DMF solvent mol­ecule [O7⋯O8: 2.658 (3) Å]. The solvent water mol­ecule donates two hydrogen bonds, one to a sulfate O atom [O10⋯O3: 2.700 (3) Å] and one to a DMF solvent mol­ecule [O10⋯O9: 2.751 (3) Å]. Within the dimeric unit, π–π inter­actions are identified between pairs of pyrazolate moieties along the sulfate-bridged sides of the trinuclear units [centroid–centroid distance: 3.641 (1) Å; dihedral angle: 7.5 (1)°].

## Database survey   

A search of the Cambridge Structural Database (Groom *et al.*, 2016 ▸) reveals only three trinuclear copper pyrazolate structures that contain sulfate (Zheng *et al.*, 2008 ▸; Di Nicola *et al.*, 2010 ▸). In all three cases, the sulfate ion coordinates weakly at the apical position of the copper cations (Cu—O bonds lengths >2.3 Å). Thus, the complex presented here is the first example of a trinuclear copper pyrazolate with the sulfate anion strongly binding at the basal position to a penta­coordinate Cu-atom [Cu1—O2: 1.976 (2) Å].

## Synthesis and crystallization   

Copper sulfate penta­hydrate (1.000 g), 4-chloro­pyrazole (411 mg) and Et_3_N (1.2 mL) were dissolved in DMF (20 mL) yielding a deep-blue solution. Dark-blue prismatic crystals of the title compound were obtained upon slow evaporation of the solvent.

## Refinement   

Crystal data, data collection and structure refinement details are summarized in Table 2 ▸. C—H hydrogen atoms were placed in idealized positions and refined using the riding-model approximation. The OH hydrogen atoms were located from difference Fourier maps; their displacement parameters were fixed to be 20% larger than those of the attached O atoms. O—H distances were restrained to 0.82 (2) Å.

## Supplementary Material

Crystal structure: contains datablock(s) I. DOI: 10.1107/S2056989016010719/gk2663sup1.cif

Structure factors: contains datablock(s) I. DOI: 10.1107/S2056989016010719/gk2663Isup2.hkl

CCDC reference: 1489622

Additional supporting information: crystallographic information; 3D view; checkCIF report

## Figures and Tables

**Table 1:** Hydrogen-bond geometry (Å, °)

*D*—H⋯*A*	*D*—H	H⋯*A*	*D*⋯*A*	*D*—H⋯*A*
C13—H13⋯O5^i^	0.93	2.23	3.155 (3)	170
C6—H6⋯O10^ii^	0.93	2.38	3.234 (4)	153
O10—H10*B*⋯O9^iii^	0.81 (2)	1.96 (2)	2.751 (3)	165 (4)
O10—H10*A*⋯O3^iv^	0.81 (2)	1.91 (2)	2.700 (3)	165 (4)
O7—H7*B*⋯O8^v^	0.83 (2)	1.83 (2)	2.658 (3)	175 (3)
O7—H7*A*⋯O10^ii^	0.80 (2)	1.83 (2)	2.625 (3)	172 (3)
O1—H1*O*⋯O9^vi^	0.78 (2)	1.95 (2)	2.711 (3)	166 (3)
O1—H1*O*⋯O9^vi^	0.78 (2)	1.95 (2)	2.711 (3)	166 (3)
O7—H7*A*⋯O10^ii^	0.80 (2)	1.83 (2)	2.625 (3)	172 (3)
O7—H7*B*⋯O8^v^	0.83 (2)	1.83 (2)	2.658 (3)	175 (3)
O10—H10*A*⋯O3^iv^	0.81 (2)	1.91 (2)	2.700 (3)	165 (4)
O10—H10*B*⋯O9^iii^	0.81 (2)	1.96 (2)	2.751 (3)	165 (4)

Symmetry codes: (i) 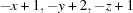; (ii) 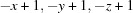; (iii) 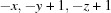; (iv) 
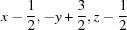
; (v) 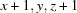; (vi) 
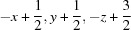
.

**Table 2:** Experimental details

Crystal data	
Chemical formula	[Cu_6_(OH)_2_(SO_4_)_2_(C_3_H_2_ClN_2_)_6_(C_3_H_7_NO)_2_(H_2_O)_2_]·4C_3_H_7_NO·2H_2_O
*M*_r_	1727.11
Crystal system, space group	Monoclinic, *P*2_1_/*n*
Temperature (K)	100
*a*, *b*, *c* (Å)	12.7038 (1), 16.5265 (2), 16.6830 (2)
β (°)	109.774 (1)
*V* (Å^3^)	3296.05 (6)
*Z*	2
Radiation type	Mo *K*α
μ (mm^−1^)	2.29
Crystal size (mm)	0.24 × 0.10 × 0.05

Data collection	
Diffractometer	Bruker APEXII CCD
Absorption correction	Multi-scan (*SADABS*; Bruker, 2014 ▸)
*T*_min_, *T*_max_	0.610, 0.894
No. of measured, independent and observed [*I* > 2σ(*I*)] reflections	39853, 8504, 6351
*R*_int_	0.061
(sin θ/λ)_max_ (Å^−1^)	0.676

Refinement	
*R*[*F* ^2^ > 2σ(*F* ^2^)], *wR*(*F* ^2^), *S*	0.035, 0.075, 1.01
No. of reflections	8504
No. of parameters	418
No. of restraints	5
H-atom treatment	H atoms treated by a mixture of independent and constrained refinement
Δρ_max_, Δρ_min_ (e Å^−3^)	0.59, −0.52

Computer programs: *APEX2* and *SAINT* (Bruker, 2014 ▸), *SHELXS97* and *SHELXTL* (Sheldrick, 2008 ▸) and *SHELXL2014* (Sheldrick, 2015 ▸).

## supplementary crystallographic information

### Crystal data

**Table:**

[Cu_6_(OH)_2_(SO_4_)_2_(C_3_H_2_ClN_2_)_6_(C_3_H_7_NO)_2_(H_2_O)_2_]·4C_3_H_7_NO·2H_2_O	*F*(000) = 1748
*M**_r_* = 1727.11	*D*_x_ = 1.740 Mg m^−^^3^
Monoclinic, *P*2_1_/*n*	Mo *K*α radiation, λ = 0.71073 Å
*a* = 12.7038 (1) Å	Cell parameters from 6640 reflections
*b* = 16.5265 (2) Å	θ = 2.6–26.9°
*c* = 16.6830 (2) Å	µ = 2.29 mm^−^^1^
β = 109.774 (1)°	*T* = 100 K
*V* = 3296.05 (6) Å^3^	Prism, blue
*Z* = 2	0.24 × 0.10 × 0.05 mm

### Data collection

**Table:**

Bruker APEXII CCD diffractometer	6351 reflections with *I* > 2σ(*I*)
φ and ω scans	*R*_int_ = 0.061
Absorption correction: multi-scan (SADABS; Bruker, 2014)	θ_max_ = 28.7°, θ_min_ = 1.8°
*T*_min_ = 0.610, *T*_max_ = 0.894	*h* = −17→17
39853 measured reflections	*k* = −20→22
8504 independent reflections	*l* = −22→22

### Refinement

**Table:**

Refinement on *F*^2^	5 restraints
Least-squares matrix: full	Hydrogen site location: mixed
*R*[*F*^2^ > 2σ(*F*^2^)] = 0.035	H atoms treated by a mixture of independent and constrained refinement
*wR*(*F*^2^) = 0.075	*w* = 1/[σ^2^(*F*_o_^2^) + (0.0294*P*)^2^ + 1.238*P*] where *P* = (*F*_o_^2^ + 2*F*_c_^2^)/3
*S* = 1.01	(Δ/σ)_max_ = 0.001
8504 reflections	Δρ_max_ = 0.59 e Å^−^^3^
418 parameters	Δρ_min_ = −0.52 e Å^−^^3^

### Special details

**Table:**

Geometry. All esds (except the esd in the dihedral angle between two l.s. planes) are estimated using the full covariance matrix. The cell esds are taken into account individually in the estimation of esds in distances, angles and torsion angles; correlations between esds in cell parameters are only used when they are defined by crystal symmetry. An approximate (isotropic) treatment of cell esds is used for estimating esds involving l.s. planes.

### Fractional atomic coordinates and isotropic or equivalent isotropic displacement parameters (Å^2^)

**Table:**

	*x*	*y*	*z*	*U*_iso_*/*U*_eq_	
Cu1	0.88533 (2)	0.98432 (2)	0.92266 (2)	0.01205 (7)	
Cu2	0.84732 (2)	0.78607 (2)	0.89552 (2)	0.01339 (8)	
Cu3	0.71256 (3)	0.89070 (2)	1.00006 (2)	0.01415 (8)	
S1	1.02182 (5)	1.14500 (4)	0.90916 (4)	0.01287 (13)	
Cl1	1.14671 (7)	0.88248 (5)	0.71858 (6)	0.0362 (2)	
Cl2	0.50045 (6)	0.57973 (5)	0.93424 (6)	0.0375 (2)	
Cl3	0.69134 (6)	1.23152 (4)	1.07089 (5)	0.02872 (17)	
O1	0.78020 (14)	0.89300 (10)	0.90866 (11)	0.0124 (4)	
H1O	0.7316 (19)	0.8995 (17)	0.8661 (13)	0.015*	
O2	1.00215 (13)	1.06890 (10)	0.95164 (11)	0.0137 (4)	
O3	0.92608 (14)	1.15827 (11)	0.83199 (12)	0.0181 (4)	
O4	1.12603 (14)	1.13455 (11)	0.89049 (12)	0.0162 (4)	
O5	1.03357 (14)	1.21179 (10)	0.96983 (11)	0.0156 (4)	
O6	0.62509 (15)	0.88937 (11)	1.07932 (12)	0.0193 (4)	
O7	0.89793 (15)	0.68175 (11)	0.86138 (13)	0.0191 (4)	
H7A	0.855 (2)	0.6528 (16)	0.8274 (16)	0.023*	
H7B	0.9493 (19)	0.6535 (16)	0.8930 (17)	0.023*	
O8	0.07007 (16)	0.59491 (12)	−0.04174 (13)	0.0265 (5)	
O9	−0.13195 (15)	0.43846 (12)	0.74393 (13)	0.0264 (5)	
O10	0.24177 (18)	0.42323 (14)	0.23718 (17)	0.0412 (7)	
H10A	0.293 (2)	0.401 (2)	0.2728 (19)	0.049*	
H10B	0.219 (3)	0.4655 (15)	0.251 (2)	0.049*	
N1	0.95307 (17)	0.92644 (13)	0.85102 (14)	0.0147 (5)	
N2	0.94773 (17)	0.84403 (13)	0.84797 (14)	0.0149 (5)	
N3	0.72096 (17)	0.73695 (13)	0.92013 (14)	0.0155 (5)	
N4	0.67343 (17)	0.77874 (13)	0.96960 (14)	0.0154 (5)	
N5	0.72004 (17)	1.00879 (13)	1.00420 (14)	0.0156 (5)	
N6	0.79401 (16)	1.04688 (13)	0.97334 (14)	0.0142 (5)	
N7	0.61860 (18)	0.90095 (14)	1.21195 (15)	0.0201 (5)	
N8	0.21667 (19)	0.62870 (14)	0.07710 (15)	0.0218 (5)	
N9	0.03464 (18)	0.37758 (13)	0.81585 (15)	0.0195 (5)	
C1	1.0125 (2)	0.81766 (16)	0.80436 (17)	0.0175 (6)	
H1	1.0244	0.7638	0.7937	0.021*	
C2	1.0585 (2)	0.88365 (17)	0.77797 (18)	0.0196 (6)	
C3	1.0202 (2)	0.95107 (17)	0.80841 (17)	0.0183 (6)	
H3	1.0378	1.0045	0.8008	0.022*	
C4	0.5943 (2)	0.73193 (16)	0.98217 (18)	0.0185 (6)	
H4A	0.5498	0.7459	1.0143	0.022*	
C5	0.5894 (2)	0.65981 (16)	0.93968 (19)	0.0207 (6)	
C6	0.6705 (2)	0.66438 (16)	0.90177 (19)	0.0201 (6)	
H6	0.6874	0.6241	0.8691	0.024*	
C7	0.7943 (2)	1.12579 (16)	0.99150 (18)	0.0175 (6)	
H7	0.8377	1.1651	0.9777	0.021*	
C8	0.7200 (2)	1.13943 (16)	1.03396 (18)	0.0192 (6)	
C9	0.6751 (2)	1.06498 (16)	1.04087 (18)	0.0187 (6)	
H9	0.6221	1.0552	1.0668	0.022*	
C10	0.6727 (2)	0.89642 (16)	1.15752 (19)	0.0199 (6)	
H10	0.7504	0.8986	1.1785	0.024*	
C11	0.4967 (2)	0.8972 (2)	1.1816 (2)	0.0321 (8)	
H11A	0.4714	0.8602	1.1347	0.048*	
H11B	0.4667	0.9500	1.1634	0.048*	
H11C	0.4719	0.8790	1.2269	0.048*	
C12	0.6772 (3)	0.9130 (2)	1.30266 (19)	0.0300 (7)	
H12A	0.6561	0.9643	1.3197	0.045*	
H12B	0.7565	0.9124	1.3138	0.045*	
H12C	0.6578	0.8705	1.3342	0.045*	
C13	0.1149 (2)	0.63767 (17)	0.02161 (19)	0.0216 (6)	
H13	0.0728	0.6803	0.0310	0.026*	
C14	0.2914 (3)	0.5656 (2)	0.0675 (2)	0.0379 (8)	
H14A	0.2527	0.5324	0.0193	0.057*	
H14B	0.3153	0.5328	0.1180	0.057*	
H14C	0.3554	0.5899	0.0589	0.057*	
C15	0.2608 (3)	0.6836 (2)	0.1482 (2)	0.0341 (8)	
H15A	0.2041	0.7218	0.1485	0.051*	
H15B	0.3241	0.7119	0.1428	0.051*	
H15C	0.2836	0.6534	0.2005	0.051*	
C16	−0.0465 (2)	0.43014 (17)	0.80778 (19)	0.0225 (6)	
H16	−0.0392	0.4640	0.8539	0.027*	
C17	0.1313 (2)	0.37298 (19)	0.89417 (19)	0.0273 (7)	
H17A	0.1206	0.4090	0.9359	0.041*	
H17B	0.1975	0.3884	0.8824	0.041*	
H17C	0.1394	0.3186	0.9156	0.041*	
C18	0.0309 (2)	0.32090 (18)	0.7482 (2)	0.0278 (7)	
H18A	−0.0347	0.3310	0.6997	0.042*	
H18B	0.0286	0.2666	0.7679	0.042*	
H18C	0.0963	0.3277	0.7324	0.042*	

### Atomic displacement parameters (Å^2^)

**Table:**

	*U*^11^	*U*^22^	*U*^33^	*U*^12^	*U*^13^	*U*^23^
Cu1	0.00914 (14)	0.01448 (16)	0.01225 (16)	−0.00096 (12)	0.00324 (12)	−0.00052 (13)
Cu2	0.01008 (14)	0.01517 (16)	0.01420 (17)	−0.00047 (12)	0.00315 (12)	−0.00099 (13)
Cu3	0.01137 (15)	0.01748 (17)	0.01439 (17)	−0.00170 (12)	0.00540 (13)	−0.00102 (13)
S1	0.0095 (3)	0.0152 (3)	0.0120 (3)	−0.0015 (2)	0.0013 (2)	0.0007 (2)
Cl1	0.0364 (4)	0.0423 (5)	0.0444 (5)	0.0025 (4)	0.0326 (4)	−0.0004 (4)
Cl2	0.0273 (4)	0.0200 (4)	0.0715 (7)	−0.0091 (3)	0.0247 (4)	−0.0017 (4)
Cl3	0.0227 (3)	0.0219 (4)	0.0440 (5)	0.0035 (3)	0.0146 (3)	−0.0092 (3)
O1	0.0079 (8)	0.0164 (9)	0.0111 (10)	−0.0004 (7)	0.0010 (7)	−0.0014 (8)
O2	0.0103 (8)	0.0159 (9)	0.0139 (10)	−0.0013 (7)	0.0025 (7)	0.0031 (7)
O3	0.0127 (9)	0.0206 (10)	0.0144 (10)	−0.0021 (7)	−0.0039 (8)	0.0042 (8)
O4	0.0118 (8)	0.0215 (10)	0.0156 (10)	−0.0023 (7)	0.0052 (8)	−0.0007 (8)
O5	0.0130 (8)	0.0163 (9)	0.0156 (10)	−0.0003 (7)	0.0024 (7)	−0.0019 (8)
O6	0.0172 (9)	0.0256 (11)	0.0172 (11)	−0.0035 (8)	0.0088 (8)	−0.0015 (8)
O7	0.0145 (9)	0.0183 (10)	0.0205 (11)	0.0018 (7)	0.0006 (8)	−0.0050 (8)
O8	0.0224 (10)	0.0286 (11)	0.0237 (12)	0.0038 (9)	0.0014 (9)	−0.0041 (9)
O9	0.0201 (10)	0.0254 (11)	0.0236 (12)	0.0033 (8)	−0.0059 (9)	−0.0051 (9)
O10	0.0244 (12)	0.0337 (14)	0.0449 (16)	0.0136 (10)	−0.0152 (11)	−0.0228 (12)
N1	0.0117 (10)	0.0180 (11)	0.0149 (12)	−0.0020 (8)	0.0053 (9)	−0.0007 (9)
N2	0.0133 (10)	0.0160 (11)	0.0149 (12)	0.0002 (8)	0.0043 (9)	−0.0030 (9)
N3	0.0117 (10)	0.0182 (12)	0.0145 (12)	−0.0015 (8)	0.0015 (9)	0.0001 (9)
N4	0.0115 (10)	0.0198 (12)	0.0159 (12)	0.0005 (9)	0.0060 (9)	0.0016 (9)
N5	0.0115 (10)	0.0185 (12)	0.0172 (12)	−0.0012 (8)	0.0054 (9)	−0.0005 (9)
N6	0.0102 (10)	0.0181 (11)	0.0142 (12)	−0.0011 (8)	0.0040 (9)	0.0005 (9)
N7	0.0206 (12)	0.0241 (13)	0.0179 (13)	0.0045 (10)	0.0094 (10)	0.0009 (10)
N8	0.0191 (12)	0.0230 (13)	0.0195 (13)	0.0034 (10)	0.0015 (10)	0.0023 (10)
N9	0.0155 (11)	0.0210 (12)	0.0177 (13)	−0.0006 (9)	−0.0002 (10)	0.0024 (10)
C1	0.0143 (12)	0.0209 (14)	0.0164 (14)	0.0012 (10)	0.0039 (11)	−0.0029 (11)
C2	0.0148 (13)	0.0284 (15)	0.0189 (15)	0.0016 (11)	0.0101 (12)	−0.0021 (12)
C3	0.0155 (13)	0.0226 (14)	0.0188 (15)	−0.0024 (11)	0.0084 (11)	0.0005 (11)
C4	0.0122 (12)	0.0206 (14)	0.0238 (16)	−0.0019 (10)	0.0075 (11)	0.0015 (11)
C5	0.0135 (12)	0.0168 (14)	0.0310 (17)	−0.0026 (10)	0.0065 (12)	0.0027 (12)
C6	0.0163 (13)	0.0164 (14)	0.0254 (16)	0.0002 (10)	0.0040 (12)	0.0000 (12)
C7	0.0131 (12)	0.0157 (13)	0.0219 (15)	0.0007 (10)	0.0036 (11)	−0.0001 (11)
C8	0.0138 (12)	0.0187 (14)	0.0248 (16)	0.0031 (10)	0.0061 (12)	−0.0033 (12)
C9	0.0150 (13)	0.0237 (15)	0.0191 (15)	0.0034 (11)	0.0077 (11)	−0.0014 (12)
C10	0.0197 (14)	0.0207 (14)	0.0226 (16)	−0.0017 (11)	0.0114 (12)	−0.0013 (12)
C11	0.0202 (15)	0.052 (2)	0.0276 (18)	0.0047 (14)	0.0125 (14)	0.0045 (15)
C12	0.0308 (16)	0.0405 (19)	0.0193 (16)	0.0059 (14)	0.0093 (13)	−0.0009 (14)
C13	0.0174 (13)	0.0245 (15)	0.0217 (16)	0.0035 (11)	0.0051 (12)	0.0018 (12)
C14	0.0251 (16)	0.039 (2)	0.043 (2)	0.0143 (14)	0.0026 (15)	0.0005 (16)
C15	0.0299 (17)	0.0330 (18)	0.0291 (19)	−0.0012 (14)	−0.0036 (14)	−0.0036 (15)
C16	0.0213 (14)	0.0230 (15)	0.0206 (16)	−0.0041 (11)	0.0035 (12)	−0.0012 (12)
C17	0.0187 (14)	0.0349 (18)	0.0221 (17)	−0.0027 (12)	−0.0013 (12)	0.0097 (13)
C18	0.0240 (15)	0.0244 (16)	0.0324 (19)	0.0040 (12)	0.0063 (14)	0.0025 (13)

### Geometric parameters (Å, º)

**Table:**

Cu1—N1	1.944 (2)	N7—C10	1.313 (3)
Cu1—N6	1.948 (2)	N7—C12	1.457 (4)
Cu1—O2	1.9760 (17)	N7—C11	1.458 (3)
Cu1—O1	1.9761 (17)	N8—C13	1.319 (3)
Cu1—O2^i^	2.2773 (17)	N8—C15	1.447 (4)
Cu2—N3	1.962 (2)	N8—C14	1.455 (4)
Cu2—N2	1.964 (2)	N9—C16	1.320 (3)
Cu2—O7	1.9895 (19)	N9—C18	1.455 (4)
Cu2—O1	2.0061 (17)	N9—C17	1.461 (3)
Cu2—O5^i^	2.2444 (18)	C1—C2	1.378 (4)
Cu3—N4	1.939 (2)	C1—H1	0.9300
Cu3—N5	1.954 (2)	C2—C3	1.380 (4)
Cu3—O1	1.9879 (18)	C3—H3	0.9300
Cu3—O6	1.9945 (18)	C4—C5	1.377 (4)
Cu3—O4^i^	2.2759 (18)	C4—H4A	0.9300
S1—O3	1.4579 (18)	C5—C6	1.382 (4)
S1—O4	1.4691 (18)	C6—H6	0.9300
S1—O5	1.4708 (18)	C7—C8	1.377 (4)
S1—O2	1.5055 (18)	C7—H7	0.9300
Cl1—C2	1.729 (3)	C8—C9	1.377 (4)
Cl2—C5	1.723 (3)	C9—H9	0.9300
Cl3—C8	1.726 (3)	C10—H10	0.9300
O1—H1O	0.775 (17)	C11—H11A	0.9600
O2—Cu1^i^	2.2774 (17)	C11—H11B	0.9600
O4—Cu3^i^	2.2759 (18)	C11—H11C	0.9600
O5—Cu2^i^	2.2444 (18)	C12—H12A	0.9600
O6—C10	1.244 (3)	C12—H12B	0.9600
O7—H7A	0.801 (17)	C12—H12C	0.9600
O7—H7B	0.831 (17)	C13—H13	0.9300
O8—C13	1.238 (3)	C14—H14A	0.9600
O9—C16	1.245 (3)	C14—H14B	0.9600
O10—H10A	0.808 (18)	C14—H14C	0.9600
O10—H10B	0.814 (18)	C15—H15A	0.9600
N1—C3	1.345 (3)	C15—H15B	0.9600
N1—N2	1.364 (3)	C15—H15C	0.9600
N2—C1	1.342 (3)	C16—H16	0.9300
N3—C6	1.345 (3)	C17—H17A	0.9600
N3—N4	1.364 (3)	C17—H17B	0.9600
N4—C4	1.340 (3)	C17—H17C	0.9600
N5—C9	1.341 (3)	C18—H18A	0.9600
N5—N6	1.368 (3)	C18—H18B	0.9600
N6—C7	1.339 (3)	C18—H18C	0.9600
			
N1—Cu1—N6	168.69 (9)	C16—N9—C18	121.7 (2)
N1—Cu1—O2	92.66 (8)	C16—N9—C17	121.1 (3)
N6—Cu1—O2	91.53 (8)	C18—N9—C17	117.2 (2)
N1—Cu1—O1	88.49 (8)	N2—C1—C2	108.7 (2)
N6—Cu1—O1	88.78 (8)	N2—C1—H1	125.7
O2—Cu1—O1	172.22 (7)	C2—C1—H1	125.7
N1—Cu1—O2^i^	96.06 (8)	C1—C2—C3	106.3 (2)
N6—Cu1—O2^i^	94.92 (8)	C1—C2—Cl1	127.0 (2)
O2—Cu1—O2^i^	82.09 (7)	C3—C2—Cl1	126.7 (2)
O1—Cu1—O2^i^	90.14 (7)	N1—C3—C2	108.4 (2)
N3—Cu2—N2	167.22 (9)	N1—C3—H3	125.8
N3—Cu2—O7	93.89 (8)	C2—C3—H3	125.8
N2—Cu2—O7	89.43 (8)	N4—C4—C5	108.9 (2)
N3—Cu2—O1	86.18 (8)	N4—C4—H4A	125.5
N2—Cu2—O1	88.44 (8)	C5—C4—H4A	125.5
O7—Cu2—O1	170.11 (8)	C4—C5—C6	106.1 (2)
N3—Cu2—O5^i^	96.85 (8)	C4—C5—Cl2	127.3 (2)
N2—Cu2—O5^i^	94.96 (8)	C6—C5—Cl2	126.6 (2)
O7—Cu2—O5^i^	97.26 (7)	N3—C6—C5	108.4 (2)
O1—Cu2—O5^i^	92.54 (7)	N3—C6—H6	125.8
N4—Cu3—N5	165.39 (9)	C5—C6—H6	125.8
N4—Cu3—O1	87.49 (8)	N6—C7—C8	109.0 (2)
N5—Cu3—O1	88.83 (8)	N6—C7—H7	125.5
N4—Cu3—O6	90.70 (8)	C8—C7—H7	125.5
N5—Cu3—O6	91.08 (8)	C7—C8—C9	105.8 (2)
O1—Cu3—O6	172.37 (7)	C7—C8—Cl3	126.3 (2)
N4—Cu3—O4^i^	96.70 (8)	C9—C8—Cl3	127.9 (2)
N5—Cu3—O4^i^	97.76 (8)	N5—C9—C8	109.2 (2)
O1—Cu3—O4^i^	96.49 (7)	N5—C9—H9	125.4
O6—Cu3—O4^i^	91.08 (7)	C8—C9—H9	125.4
O3—S1—O4	111.90 (11)	O6—C10—N7	123.2 (3)
O3—S1—O5	110.78 (11)	O6—C10—H10	118.4
O4—S1—O5	110.20 (10)	N7—C10—H10	118.4
O3—S1—O2	108.64 (10)	N7—C11—H11A	109.5
O4—S1—O2	107.86 (10)	N7—C11—H11B	109.5
O5—S1—O2	107.30 (10)	H11A—C11—H11B	109.5
Cu1—O1—Cu3	111.97 (8)	N7—C11—H11C	109.5
Cu1—O1—Cu2	112.98 (8)	H11A—C11—H11C	109.5
Cu3—O1—Cu2	112.15 (8)	H11B—C11—H11C	109.5
Cu1—O1—H1O	107 (2)	N7—C12—H12A	109.5
Cu3—O1—H1O	107 (2)	N7—C12—H12B	109.5
Cu2—O1—H1O	105 (2)	H12A—C12—H12B	109.5
S1—O2—Cu1	134.93 (11)	N7—C12—H12C	109.5
S1—O2—Cu1^i^	127.14 (10)	H12A—C12—H12C	109.5
Cu1—O2—Cu1^i^	97.91 (7)	H12B—C12—H12C	109.5
S1—O4—Cu3^i^	118.91 (11)	O8—C13—N8	126.4 (3)
S1—O5—Cu2^i^	125.29 (10)	O8—C13—H13	116.8
C10—O6—Cu3	120.80 (17)	N8—C13—H13	116.8
Cu2—O7—H7A	121 (2)	N8—C14—H14A	109.5
Cu2—O7—H7B	125 (2)	N8—C14—H14B	109.5
H7A—O7—H7B	108 (3)	H14A—C14—H14B	109.5
H10A—O10—H10B	118 (4)	N8—C14—H14C	109.5
C3—N1—N2	108.4 (2)	H14A—C14—H14C	109.5
C3—N1—Cu1	131.89 (19)	H14B—C14—H14C	109.5
N2—N1—Cu1	119.18 (16)	N8—C15—H15A	109.5
C1—N2—N1	108.2 (2)	N8—C15—H15B	109.5
C1—N2—Cu2	131.42 (18)	H15A—C15—H15B	109.5
N1—N2—Cu2	120.19 (15)	N8—C15—H15C	109.5
C6—N3—N4	108.4 (2)	H15A—C15—H15C	109.5
C6—N3—Cu2	132.94 (19)	H15B—C15—H15C	109.5
N4—N3—Cu2	118.57 (16)	O9—C16—N9	125.8 (3)
C4—N4—N3	108.2 (2)	O9—C16—H16	117.1
C4—N4—Cu3	130.49 (18)	N9—C16—H16	117.1
N3—N4—Cu3	121.01 (15)	N9—C17—H17A	109.5
C9—N5—N6	107.8 (2)	N9—C17—H17B	109.5
C9—N5—Cu3	133.20 (18)	H17A—C17—H17B	109.5
N6—N5—Cu3	118.53 (16)	N9—C17—H17C	109.5
C7—N6—N5	108.2 (2)	H17A—C17—H17C	109.5
C7—N6—Cu1	131.22 (18)	H17B—C17—H17C	109.5
N5—N6—Cu1	120.33 (16)	N9—C18—H18A	109.5
C10—N7—C12	121.5 (2)	N9—C18—H18B	109.5
C10—N7—C11	120.0 (2)	H18A—C18—H18B	109.5
C12—N7—C11	118.4 (2)	N9—C18—H18C	109.5
C13—N8—C15	121.4 (2)	H18A—C18—H18C	109.5
C13—N8—C14	121.6 (3)	H18B—C18—H18C	109.5
C15—N8—C14	116.9 (2)		

Symmetry code: (i) −*x*+2, −*y*+2, −*z*+2.

### Hydrogen-bond geometry (Å, º)

**Table:**

*D*—H···*A*	*D*—H	H···*A*	*D*···*A*	*D*—H···*A*
C18—H18*B*···O3^ii^	0.96	2.64	3.494 (4)	148
C17—H17*C*···O5^ii^	0.96	2.56	3.360 (3)	141
C15—H15*C*···N2^iii^	0.96	2.63	3.406 (4)	138
C13—H13···O5^iv^	0.93	2.23	3.155 (3)	170
C10—H10···O4^i^	0.93	2.30	2.971 (3)	128
C7—H7···O5	0.93	2.65	3.483 (3)	149
C7—H7···S1	0.93	2.95	3.610 (3)	129
C6—H6···O10^v^	0.93	2.38	3.234 (4)	153
C4—H4*A*···Cl3^vi^	0.93	2.93	3.484 (3)	119
C3—H3···O4	0.93	2.64	3.411 (3)	140
C3—H3···S1	0.93	2.99	3.616 (3)	126
O10—H10*B*···O9^vii^	0.81 (2)	1.96 (2)	2.751 (3)	165 (4)
O10—H10*A*···O3^iii^	0.81 (2)	1.91 (2)	2.700 (3)	165 (4)
O7—H7*B*···O8^viii^	0.83 (2)	1.83 (2)	2.658 (3)	175 (3)
O7—H7*A*···O10^v^	0.80 (2)	1.83 (2)	2.625 (3)	172 (3)
O1—H1*O*···O9^ix^	0.78 (2)	1.95 (2)	2.711 (3)	166 (3)
O1—H1*O*···O9^ix^	0.78 (2)	1.95 (2)	2.711 (3)	166 (3)
O7—H7*A*···O10^v^	0.80 (2)	1.83 (2)	2.625 (3)	172 (3)
O7—H7*B*···O8^viii^	0.83 (2)	1.83 (2)	2.658 (3)	175 (3)
O10—H10*A*···O3^iii^	0.81 (2)	1.91 (2)	2.700 (3)	165 (4)
O10—H10*B*···O9^vii^	0.81 (2)	1.96 (2)	2.751 (3)	165 (4)
C3—H3···S1	0.93	2.99	3.616 (3)	126
C3—H3···O4	0.93	2.64	3.411 (3)	140
C4—H4*A*···Cl3^vi^	0.93	2.93	3.484 (3)	119
C6—H6···O10^v^	0.93	2.38	3.234 (4)	153
C7—H7···S1	0.93	2.95	3.610 (3)	129
C7—H7···O5	0.93	2.65	3.483 (3)	149
C10—H10···O4^i^	0.93	2.30	2.971 (3)	128
C13—H13···O5^iv^	0.93	2.23	3.155 (3)	170
C15—H15*C*···N2^iii^	0.96	2.63	3.406 (4)	138
C17—H17*C*···O5^ii^	0.96	2.56	3.360 (3)	141
C18—H18*B*···O3^ii^	0.96	2.64	3.494 (4)	148

Symmetry codes: (i) −*x*+2, −*y*+2, −*z*+2; (ii) *x*−1, *y*−1, *z*; (iii) *x*−1/2, −*y*+3/2, *z*−1/2; (iv) −*x*+1, −*y*+2, −*z*+1; (v) −*x*+1, −*y*+1, −*z*+1; (vi) −*x*+1, −*y*+2, −*z*+2; (vii) −*x*, −*y*+1, −*z*+1; (viii) *x*+1, *y*, *z*+1; (ix) −*x*+1/2, *y*+1/2, −*z*+3/2.
